# An improved YOLOv8-seg-based method for key part segmentation of tobacco plants

**DOI:** 10.3389/fpls.2025.1673202

**Published:** 2025-09-22

**Authors:** Yihao Liu, Du Chen, Yawei Zhang, Xin Wang

**Affiliations:** 1 College of Engineering, China Agricultural University, Beijing, China; 2 State Key Laboratory of Intelligent Agricultural Power Equipment, Beijing, China

**Keywords:** YOLOv8, deep learning, agricultural robots, tobacco harvesting, automated harvesting

## Abstract

Accurate segmentation of key tobacco structures is essential for enabling automated harvesting. However, complex backgrounds, variable lighting conditions, and blurred boundaries between the stem and petiole significantly hinder segmentation accuracy in field environments. To overcome these challenges, we propose an enhanced instance segmentation approach based on YOLOv8-seg, incorporating depth-based background filtering and architectural improvements. Specifically, depth information from RGB-D images is employed to spatially filter non-target background regions, thereby enhancing foreground clarity. In addition, a Hybrid Dilated Residual Attention Block (HDRAB) is integrated into the YOLOv8-seg backbone to improve boundary discrimination between petioles and stems, while a Lightweight Shared Detail-Enhanced Convolution Detection Head (LSDECD) is designed to efficiently capture fine-grained texture features. Experimental results demonstrate that depth filtering increases mAP50^bb^ and mAP50^seg^ by 7.9% and 6.3%, respectively, while the architectural enhancements further raise them to 89.5% and 91.1%, surpassing the YOLOv8-seg baseline by 5.2% and 10.0%. Compared with mainstream models such as Mask R-CNN and SOLOv2, the proposed method achieves superior segmentation accuracy with low computational cost, highlighting its potential for practical deployment in automated tobacco harvesting

## 1 Introduction

Tobacco is one of China’s most important cash crops, accounting for approximately half of the world’s total production (Tang et al., 2020; Shen et al., 2021). However, the harvesting of tobacco leaves remains heavily reliant on manual labor, leading to high labor costs and an increasingly aging workforce (Lencucha et al., 2022; Wang et al., 2023). Consequently, the development of robotic systems capable of automating high-intensity tobacco leaf harvesting has become an urgent priority (Thakur et al., 2023). A fundamental prerequisite for intelligent harvesting is the accurate perception of key structural components of the plant. Therefore, establishing a robust method for segmenting critical parts of tobacco—specifically the petioles and main stems—under unstructured field conditions is of great significance for advancing research on automated tobacco harvesting robots (Zhou et al., 2022).

**Figure 1:**
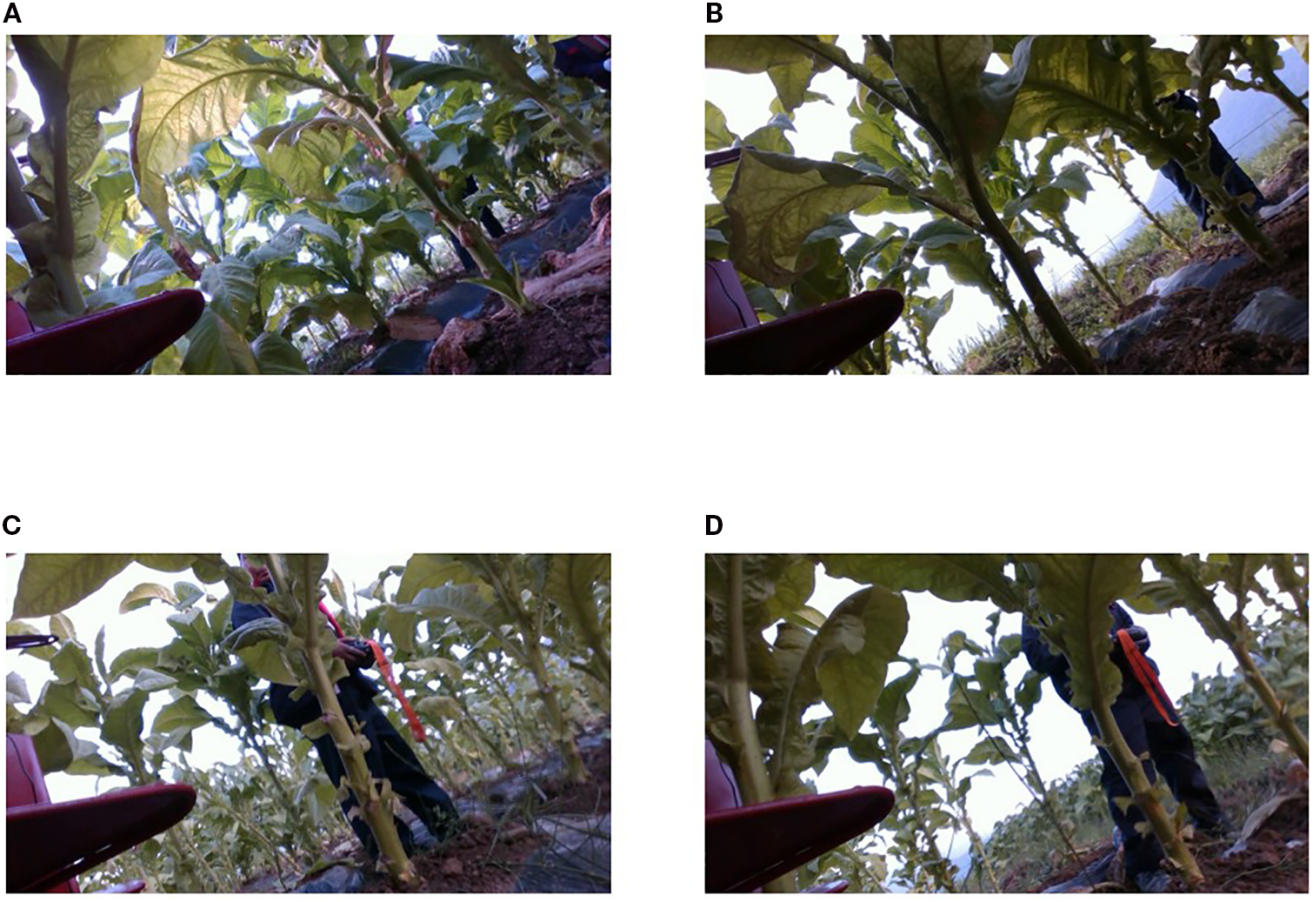
Tobacco images under different lighting conditions. **(A)** Sunny day with direct light. **(B)** Sunny day with backlight. **(C)** Cloudy day with direct light. **(D)** Cloudy day with backlight.

Most existing tobacco detection and segmentation methods have primarily focused on phenotypic analysis (Li et al., 2022; Zhang et al., 2023), maturity assessment of tobacco leaves (Lu et al., 2023; Sun et al., 2023; Dai et al., 2024), pest and disease identification, and weed detection (Zhu et al., 2017; Tufail et al., 2021), rather than on automated harvesting. However, the unique challenges posed by tobacco harvesting in complex field environments remain largely unaddressed. Due to the growth characteristics of tobacco plants, the petiole and main stem often appear within the same side of the camera’s field of view during data collection—resulting in frequent occlusions(with the petiole occluding the stem). These occlusion scenarios account for approximately 60% of all dataset instances (**Figures 1B, D**), contrasting with cases where petioles grow on lateral branches of the stem (**Figure 1A**). Furthermore, the petiole and main stem often share similar coloration, making it difficult to distinguish clear boundaries and adversely impacting segmentation accuracy. The criteria for distinguishing between these two structures are detailed in Section II.B.

In addition to structural occlusions, variability in lighting conditions poses further challenges for segmenting key tobacco structures. Under strong backlighting (**Figure 1D**), for example, low illumination at the junction between the petiole and stem leads to blurred contours, reducing detection model performance. Yue et al. (2023) noted that illumination changes under natural light can easily lead to segmentation errors in agricultural vision tasks. As shown in **Figure 1**, the structural textures of petioles vary significantly under different lighting conditions, and standard convolutional architectures often struggle to consistently extract such heterogeneous features. Therefore, enhancing the model’s ability to perceive fine-grained texture details under variable illumination is essential.

To address these challenges, many studies have incorporated structural enhancement modules such as attention mechanisms and multi-scale convolutions (Xu et al., 2022; Zhang et al., 2023; Guo et al., 2024). For instance, Shen et al. (2025) proposed the MSA-YOLO model, introducing the Multi-scale Attention Mixing Head (MAMH) to improve the segmentation of grape pedicels from visually similar background elements. Wang et al. Qingyi et al. (2025) developed the CS-YOLO network to tackle issues of indistinct crack boundaries and scale variations, enhancing detection of subtle textures through high-dimensional feature mapping and multi-layer feature fusion. In another study, Wang et al. (2024) proposed an improved NVW-YOLOv8s network featuring a residual feature learning module based on a Normalization-based Attention Module (NAM) to address occlusions and boundary ambiguities in fruit segmentation.

At present, many researchers have focused on multimodal detection and segmentation methods, such as infrared-based few-shot object detection (Zhang et al., 2025b), visible-infrared person re-identification (Zhang et al., 2025a), and RGB-D salient object detection (Zhang et al., 2024). These studies highlight the effectiveness of multimodal feature fusion in enhancing robustness under diverse conditions. More recently, multimodal strategies for land cover interpretation, such as diffusion model–based remote sensing change captioning frameworks (Sun et al; Pang et al., 2025), further underscore the effectiveness of cross-modal integration in complex perception tasks. However, compared with relatively stable industrial or surveillance environments, agricultural scenarios are more complex and dynamic, requiring solutions that address challenges such as occlusion, illumination variation, and structural ambiguity. For instance, Li et al. (2022) proposed PSegNet, which performs semantic and instance segmentation on plant point clouds, demonstrating robust structural discrimination. More recently, Xie et al. (2025) developed a temporal semantic multispectral (TSM) point cloud generation and feature fusion pipeline for greenhouse tomatoes, integrating RGB-D and multispectral imaging to enable comprehensive trait estimation. Although these point cloud or multimodal strategies provide highly detailed structural and spectral information, they often require sophisticated imaging systems, multi-step registration, and radiometric calibration, which considerably increase the cost and limit scalability in field applications. In contrast, the RGB-D perception strategy adopted in this study achieves a more cost-effective and practical balance, offering sufficient structural cues while maintaining feasibility for deployment in real-world tobacco harvesting scenarios.

Although structural enhancement modules can significantly improve model accuracy, they often come at the cost of increased parameter counts and computational demands. Consequently, recent research has also focused on lightweight crop segmentation models suitable for deployment on edge devices (Wang and He, 2021). For example, Paul et al. (2024) applied channel pruning techniques to the YOLOv8s network to reduce model complexity while achieving effective segmentation performance. However, such lightweight designs may come at the expense of reduced segmentation accuracy.

In summary, existing segmentation models primarily emphasize distinguishing targets from complex backgrounds but pay limited attention to differentiating internal structural components, such as the junction between the petiole and main stem in tobacco. Moreover, achieving a balance between high segmentation accuracy and low computational complexity remains a critical challenge for real-time and edge deployment. Thus, there is a pressing need for segmentation approaches specifically designed to address the structural intricacies of tobacco plants while maintaining computational efficiency.

To address the aforementioned challenges, this study proposes an enhanced instance segmentation approach based on YOLOv8-seg, incorporating depth information preprocessing and architectural optimization. Firstly, RGB-D images captured by a depth camera are utilized in conjunction with a background depth thresholding strategy to filter out non-target regions, thereby substantially improving foreground segmentation quality. Next, a Hybrid Dilated Residual Attention Block (HDRAB) is integrated into the YOLOv8 backbone, combining hybrid dilated convolutions with channel attention mechanisms to enhance the model’s ability to differentiate between petiole and stem boundaries under varying lighting conditions. Finally, to further reduce computational overhead, a lightweight detection head, termed LSDECD (Lightweight Shared Detail-Enhanced Convolution Detection Head), is developed by sharing convolutional layers between the regression and classification branches to reduce computational overhead, while introducing multi-directional differential convolutions (DEConv) to improve the extraction of fine-grained local features. The proposed method in this paper establishes a robust perceptual foundation for automated tobacco harvesting and structural analysis.

## 2 Materials and methods

### 2.1 Data acquisition

**Figure 2:**
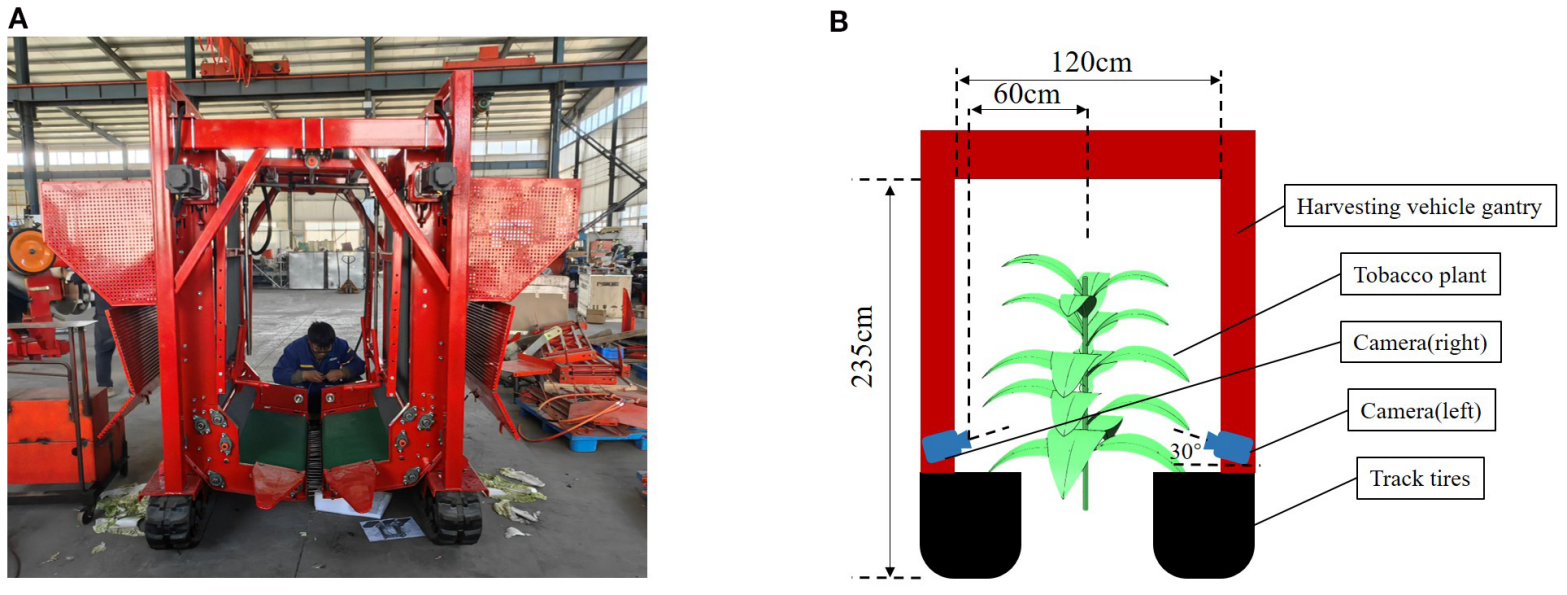
Data collection diagram. **(A)** Photograph of the harvesting vehicle. **(B)** Schematic of the sensor installation.

Data for this study were collected in April, 2024, in a tobacco field located in Wengjiao Village, Mangshi City, Dehong Dai and Jingpo Autonomous Prefecture, Yunnan Province, China (24°22′21″N, 98°33′13″E). An RGB-D camera (Realsense D435 (Intel RealSense, 2025)) was mounted on a tobacco leaf harvester, as illustrated in **Figure 2A**, to capture the dataset. The harvester is equipped a gantry measuring 235 cm in height and 120 cm in width, enabling it to straddle crop ridges and move directly above the tobacco plants. To enhance operational stability under field conditions, crawler-type tires were installed. The camera was mounted beneath the front section of the gantry, aligned in the direction of travel, and positioned at a downward tilt angle of 30° relative to the horizontal plane. Continuous video recording was employed for data acquisition, with videos recorded at a resolution of 1280×720 pixels. The camera was maintained at an approximate distance of 50~60 cm from the main stem of the plants, ensuring operation well within the effective depth range of the D435 sensor. A schematic of the installation setup is shown in **Figure 2B**. Following recording, the video footage was segmented into still frames for further processing.

To ensure diversity in lighting conditions, data were collected under both sunny and cloudy skies, with forward and backlighting scenarios represented, as shown in **Figure 1**. All images utilized in this study were acquired from the right-side camera. A total of 2,366 tobacco images were extracted from the video footage, including 692 images under sunny backlighting, 455 under sunny front lighting, 522 under cloudy backlighting, and 697 under cloudy front lighting. The dataset was split into training, validation, and test sets in a ratio of 8:1:1.

### 2.2 Annotation strategy

**Figure 3:**
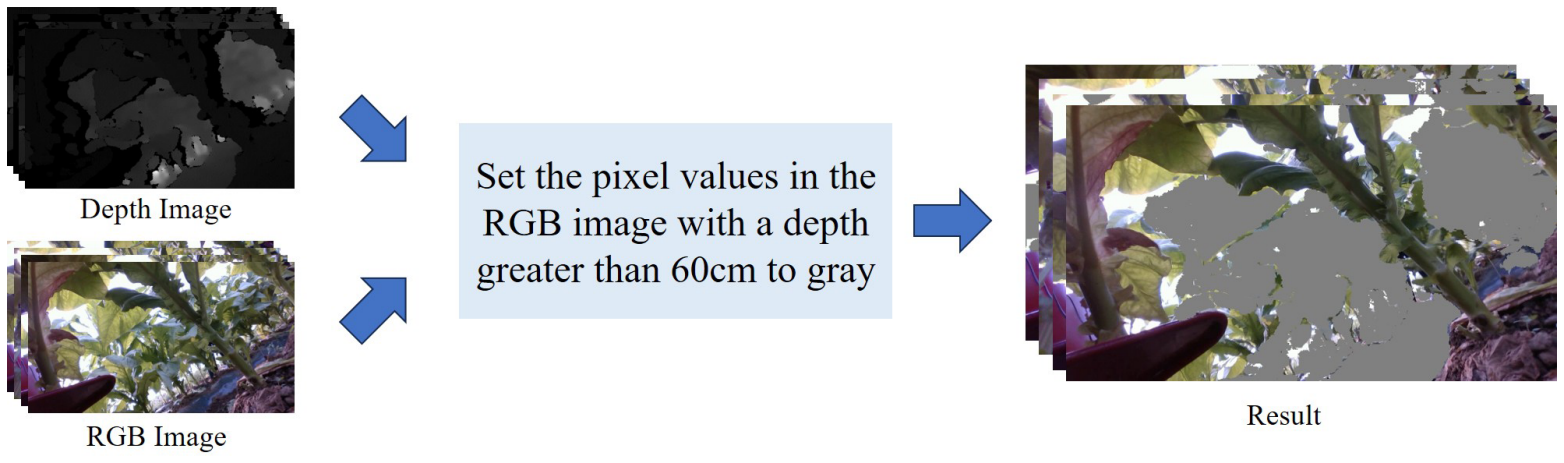
Depth filtering diagram.

Due to the dense growth of tobacco plants and the presence of similarly colored vegetation in the background, segmentation of key structural parts is prone to interference. Previous studies have demonstrated that background filtering can improve detection performance (Arsenovic et al., 2019). To address this issue, a depth-based background filtering approach was employed in this study: pixels with depth values greater than 60 cm were reassigned a grayscale value of 128, thereby suppressing interference from background regions with similar coloration. An illustration of this process is provided in **Figure 3**.

**Figure 4:**
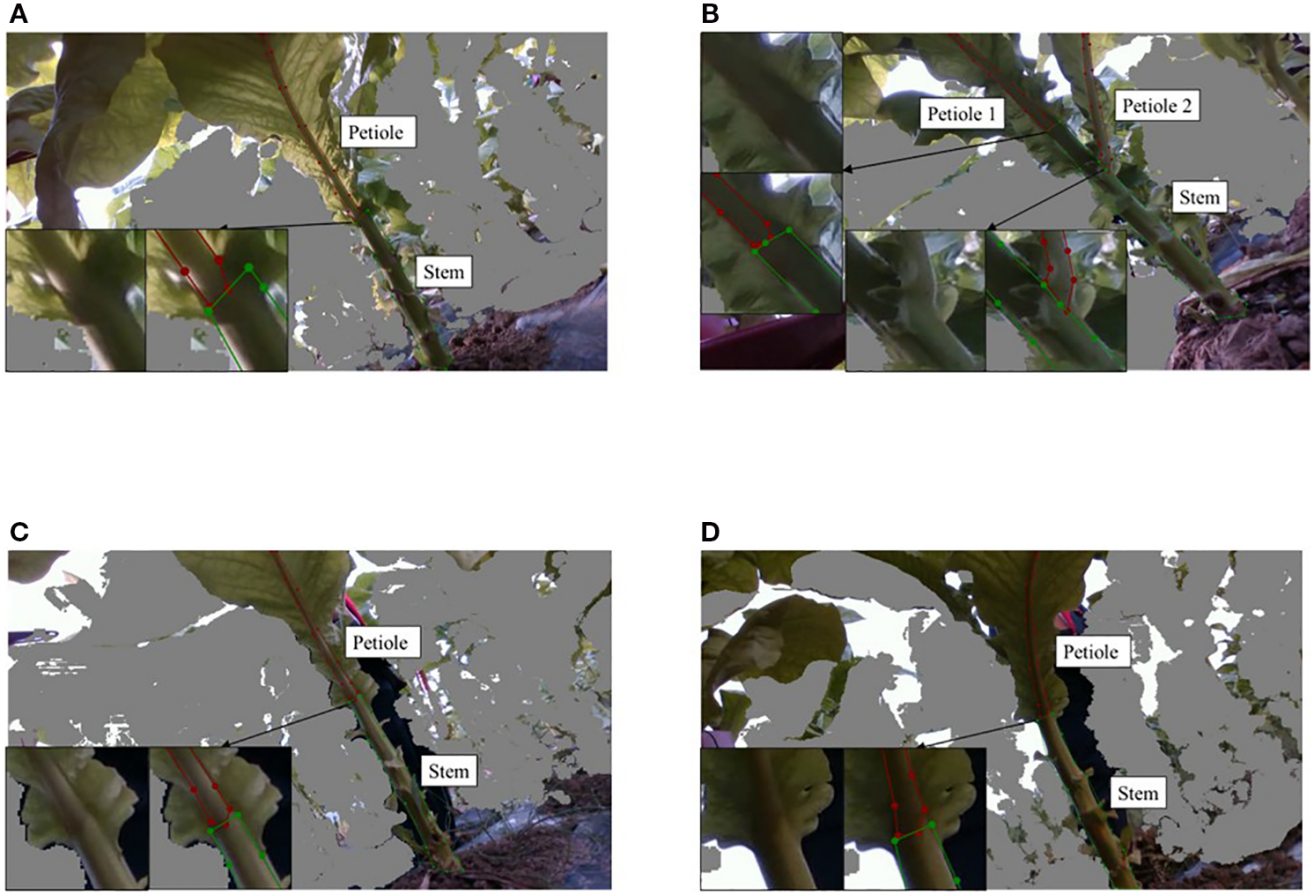
Annotation strategy for key parts of tobacco. **(A)** Sunny day with direct light. **(B)** Sunny day with backlight. **(C)** Cloudy day with direct light. **(D)** Cloudy day with backlight.

Following depth filtering, the tobacco images were manually annotated using the Labelme tool, resulting in labels in JSON format. These annotations were subsequently converted into YOLO-compatible.txt files. The labeling strategy is depicted in **Figure 4**. For each petiole–stem pair, the growth node was used as a reference point. From this node, the visible portion of the petiole (including leaf veins) within the camera’s field of view was annotated, as shown in red in **Figure 4**. The main stem was annotated from the ground up to the point where it becomes occluded by the petiole, as annotated in green. Only unobstructed regions were labeled to ensure annotation consistency. To enhance boundary clarity, magnified insets focusing on the junctions between petioles and stems are provided in the lower left corner of **Figure 4**.

During training, automatic data augmentations were applied, expanding the size of the training set by approximately tenfold and thereby mitigating the risk of model overfitting.

### 2.3 Model improvements

YOLO is a state-of-the-art single-stage object detection model that has been widely adopted in both industrial and agricultural applications due to its excellent performance (Zhou et al., 2022; Badgujar et al., 2024). YOLOv8, developed by the Ultralytics team in collaboration with numerous contributors, builds upon the YOLOv5 framework with extensive architectural improvements to support real-time, multi-task learning. It further improves model accuracy and generalization capabilities, while significantly outperforms earlier models in terms of deployment efficiency on edge devices. In this study, YOLOv8-seg is selected as the baseline model for further improvement and comparison. At the time our work was initiated, YOLOv8-seg was the latest and most stable segmentation framework available, widely adopted in the community. Although newer versions such as YOLOv9–YOLOv13 have since been released, their segmentation branches have not yet been as mature or reproducible, with most updates focusing on detection rather than instance segmentation. Therefore, YOLOv8-seg provides a more reliable and practical baseline for enhancement in the context of tobacco plant segmentation.

#### 2.3.1 Multi-scale attention-based structural enhancement module

**Figure 5:**
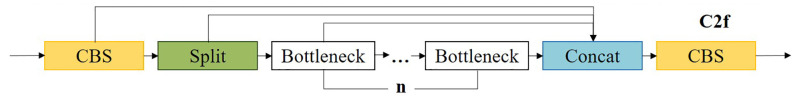
C2f structure diagram.

In YOLOv8 architecture, the C2f module (CSP Bottleneck with Two Convolutions and Fusion) serves as a key component for feature extraction. It is an improved version of the Cross Stage Partial (CSP) structure, specifically designed to enhance feature extraction and fusion efficiency. Compared to the traditional CSP architecture, C2f further reduces computational costs, enabling YOLOv8 to achieve a more effective balance between lightweight design and high performance. By integrating direct connections for a subset of features with bottleneck-style transformations for the remainder, C2f improves computational efficiency while preserving strong feature representation capabilities. A schematic illustration of the C2f architecture is provided in **Figure 5**.

**Figure 6:**
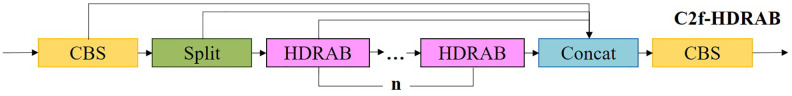
C2f-HDRAB structure diagramThe Hybrid Dilated Residual Attention Block (HDRAB) consists of two components: a hybrid dilated residual block and a Channel Attention Module (CAM), as illustrated in **Figure 7**. The hybrid dilated residual block comprises multiple hybrid dilated convolutions (denoted as n-DConv in **Figure 7**) with ReLU activations. These dilated convolutions are connected via multiple skip connections, enabling the capture of rich local features. Here, *n* represents the dilation rate, ranging from 1 to 4, and the “⊕” symbol denotes element-wise addition, which facilitates aggregation of spatial information across scales.

Traditional bottleneck modules typically employ 3×3 convolutions for feature extraction; however, their limited receptive fields constrain the ability to capture global contextual information. To enhance the capability of YOLOv8-seg in segmenting complex key parts of tobacco plants, the bottleneck component within the C2f structure was replaced with a Hybrid Dilated Residual Attention Block (HDRAB), as shown in **Figure 6**. Unlike conventional bottlenecks, HDRAB integrates hybrid dilated convolutions with residual attention mechanisms, facilitating multi-scale feature fusion and significantly improving the model’s capacity to capture spatial details across varying scales. This architectural enhancement contributes to better instance segmentation performance in complex agricultural scenarios (Wu et al., 2024).

**Figure 7:**
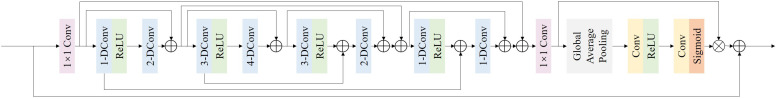
The architecture of the HDRAB.

The Channel Attention Module (CAM) is composed of Global Average Pooling (GAP), a convolutional layer, ReLU, and a Sigmoid activation function. CAM is used to explore the inter-channel dependencies among convolutional features. In **Figure 7**, the “⊗” symbol represents element-wise multiplication, representing channel-wise reweighting.

To further optimize the original HDRAB, two additional 1×1 convolutions before and after each dilated convolution (DConv), as shown in pink in **Figure 7**. The first 1×1 convolution reduces the feature dimensionality, while the second restores the number of channels. This pre-reduction and post-restoration strategy reduces computational cost while preserving feature integrity. Moreover, it enables more efficient feature processing within the DConv layers, improving information flow and achieving a better balance between computational efficiency and object detection accuracy.

In practice, tobacco petioles and stems often exhibit blurred or overlapping boundaries, especially under complex lighting. The HDRAB structure was therefore designed to enlarge the receptive field while preserving spatial resolution, enabling the network to capture both global context and fine boundary cues. By coupling residual connections with channel attention, HDRAB selectively strengthens boundary-relevant features and mitigates the ambiguity at stem–petiole junctions.

#### 2.3.2 Improved lightweight detection head

**Figure 8:**
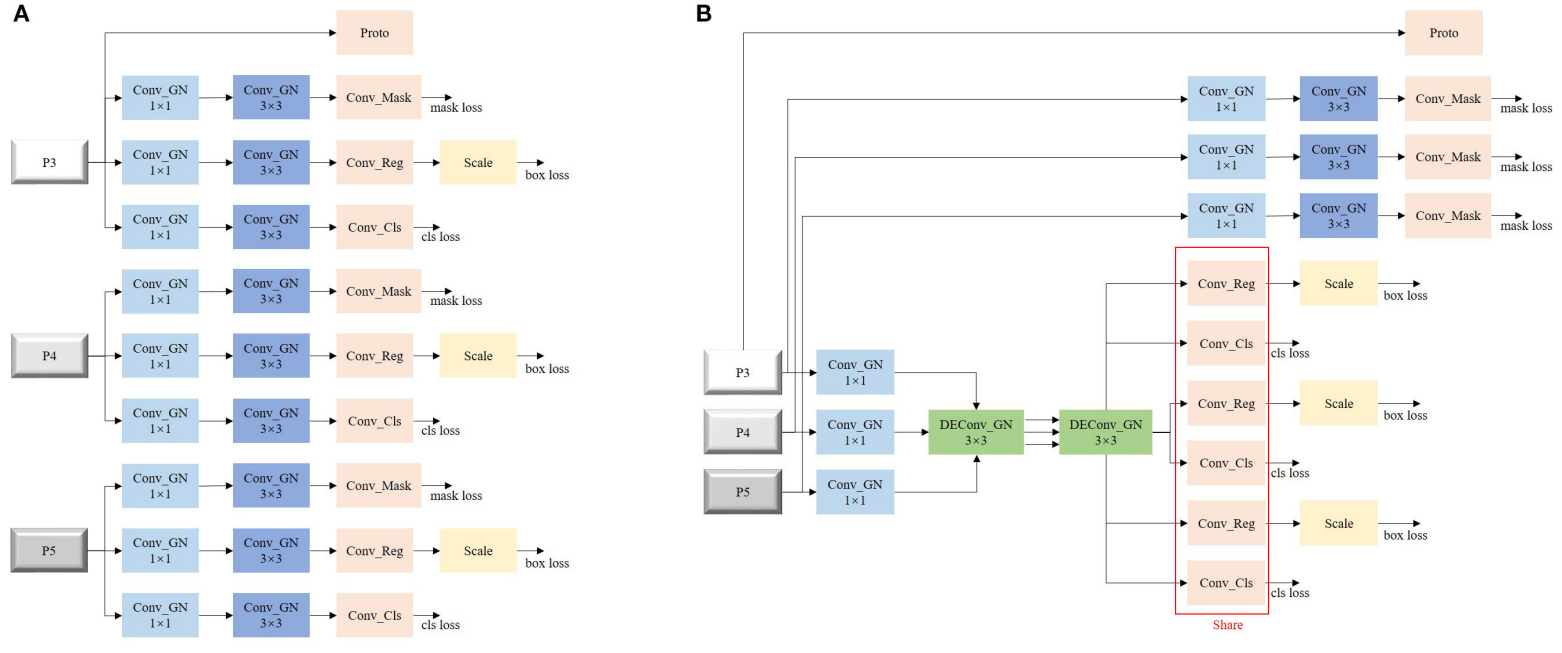
Detection head. **(A)** original detection head. **(B)** improved detection head.

The detection head in YOLOv8-seg adopts a multi-scale feature fusion strategy, performing object detection and instance segmentation on feature maps at 3 different scales—P3, P4, and P5—as illustrated in **Figure 8A**. Each feature map first undergoes dimensionality reduction through a 1×1 convolution to decrease computational cost and enhance feature expressiveness, followed by a 3×3 convolution for further local feature extraction.

Based on this structure, the detection head is divided into three task-specific branches: a mask branch, a regression branch, and a classification branch. In YOLOv8-seg, the mask branch operates on top of detection outputs, such that improvements in detection accuracy directly enhance segmentation performance.

To further optimize this process, we propose a novel detection head, termed Lightweight Shared Detail-Enhanced Convolution Detection Head (LSDECD), as shown in **Figure 8B**, to replace the original design. In LSDECD, the 3×3 convolutions in the regression and classification branches across P3, P4, and P5 are shared, effectively reducing computational load. Given the structural randomness in the growth of key tobacco components in unstructured environments, multi-directional differential convolutions (DEConv) are employed as the shared 3×3 convolution layers to improve fine-grained detail extraction and enhance the accuracy of the lightweight detection head. Since instance segmentation demands high-resolution boundary predictions, the Conv_mask module is not shared with other branches, thereby preserving the segmentation precision.

**Figure 9:**
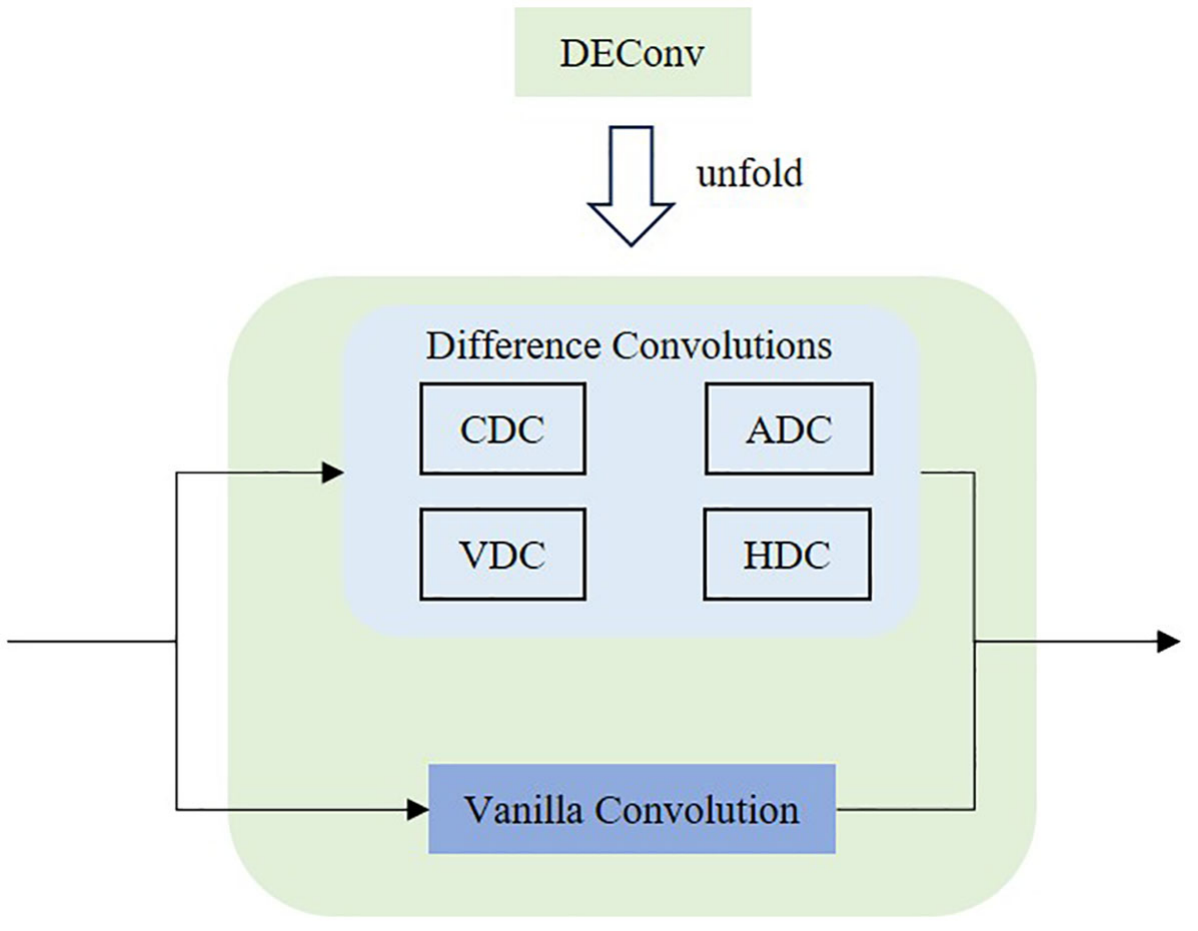
The architecture of the DEConv.

The full name of DEConv is Detail-Enhanced Convolution (Chen et al., 2024), which is specifically designed to enhance the detection head’s ability to extract fine-grained details, particularly in response to the unstructured growth patterns of tobacco plants. As shown in **Figure 9**, DEConv consists of five parallel convolutional layers: one standard (vanilla) convolution layer and four difference-based convolution layers. These four layers are: Central Difference Convolution (CDC), Angular Difference Convolution (ADC), Horizontal Difference Convolution (HDC), and Vertical Difference Convolution (VDC). Each is responsible for extracting local gradient information in a specific direction, enabling the network to better capture high-frequency features such as edges and texture details.

DEConv exhibits distinctive behavior in convolutional operations. When multiple convolutional kernels with identical size, stride, and padding are applied in parallel to the same input, the sum of their outputs is mathematically equivalent to summing the kernels first and then performing a single convolution. As a result, DEConv does not increase the number of parameters or computational cost, making it well-suited for lightweight applications. Given an input feature map, the output of DEConv can be expressed as Equation 1:

(1) $$F_{out}=\sum_{i=1}^{5}F_{in}*K_{i}=F_{in}*K_{cut}$$

where $K_{i}(i=1:5)$ represents the five individual convolution kernels, ∗ denotes the convolution operation, and $K_{cut}$ is the equivalent kernel obtained by summing the parallel kernels. DEConv enables efficient extraction of fine-grained image features such as texture, shape, and color, making it particularly effective for meeting the detailed requirements of instance segmentation tasks.

Considering the need for efficient field deployment, the LSDECD head integrates shared convolutional layers to reduce redundancy between classification and regression branches. At the same time, the DEConv module enhances sensitivity to local high-frequency gradients, which is critical for distinguishing slender petioles from occluding leaves. This combination allows the model to achieve fine-grained feature extraction while maintaining lightweight computation.

#### 2.3.3 YOLOv8 network overall structure

To enhance the model’s structural perception of key tobacco components, a structural enhancement module—Hybrid Dilated Residual Attention Block (HDRAB)—is introduced, combining multi-scale dilated convolutions with a channel attention mechanism. While replacing all C2f modules with HDRAB could improve feature extraction capabilities, such extensive modification would inevitably degrade inference speed and hinder deployment on field devices. Therefore, a selective modification strategy is adopted based on the specific requirements of key-part segmentation in tobacco plants.

In the YOLOv8 backbone, the shallow C2f modules primarily extract low-level features such as edges and textures. Introducing attention mechanisms at this stage may overemphasize irrelevant channels and lead to overfitting on redundant features. Thus, the original C2f structures are retained in the shallow layers. In contrast, the mid-to-deep layers (Stages 3 to 5) are critical for semantic aggregation and for modeling the spatial relationships between the tobacco stem and petiole. Accordingly, HDRAB modules are embedded into these stages to enhance the model’s capacity for capturing structural continuity and non-local semantic dependencies.

**Figure 10:**
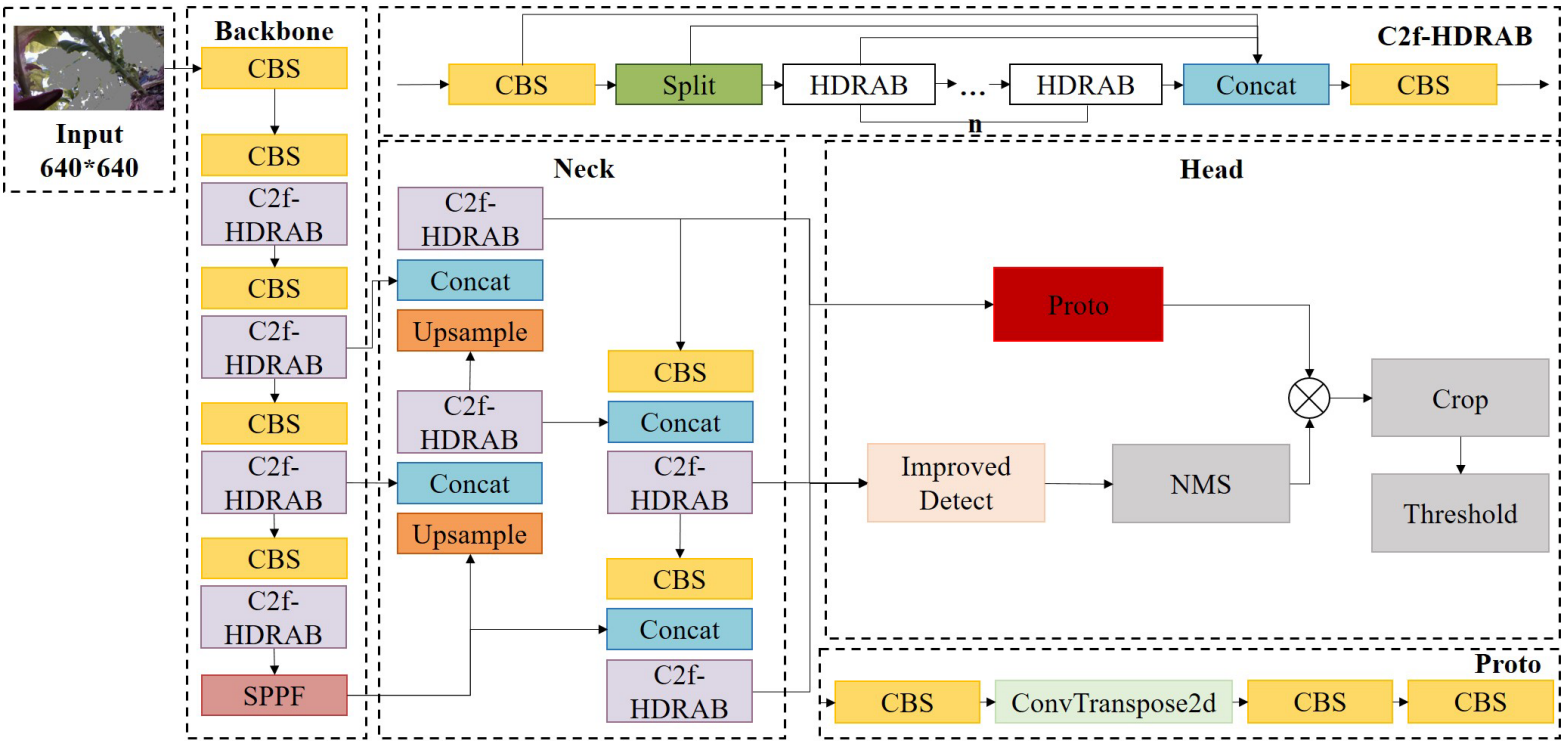
Improved YOLOv8-seg model architecture.

Within the network’s neck, HDRAB is not applied to the downsampling path to maintain computational efficiency. However, HDRAB modules are incorporated into the upsampling path to reinforce boundary detail representation in high-resolution feature maps. This modification is particularly beneficial near the output layers—such as the P3 level—where the non-local modeling capability of HDRAB significantly improves the distinction between the petiole and the main vein. Furthermore, in the final C2f module before the detection head output—where semantic features are densely fused and exert substantial influence on segmentation accuracy—HDRAB is also introduced to further enhance structural feature representation. The complete integration strategy is illustrated in **Figure 10**.

### 2.4 Experimental conditions

All model training and testing were conducted on a desktop workstation with the following hardware configuration: Windows 10 operating system, Intel Core i5-13490F CPU, NVIDIA GeForce RTX 4060 Ti GPU, and 32 GB of RAM. The software environment included PyTorch 2.0.0, CUDA 11.8, and Python 3.9. The model was trained for 300 epochs with an input resolution of 640 × 640 and a batch size of 4. We employed the SGD optimizer with an initial learning rate of 0.01, momentum of 0.937, and weight decay of 0.0005, following the default YOLOv8 training strategy.

### 2.5 Evaluation indicators

Given the importance of accurate mask and localization performance in evaluating segmentation models, we adopt both bounding box mean Average Precision (mAP) and mask mAP as the primary evaluation metrics for key part segmentation in this study. Two types of Intersection over Union (IoU) thresholds are used: a fixed threshold of 0.5, denoted as mAP50, and a range from 0.5 to 0.95 (in increments of 0.05), averaged and referred to as mAP50–95. A predicted bounding box is considered a true positive if its IoU with the ground truth polygon exceeds the specified threshold. Otherwise, it is treated as a false positive. If no predicted box meets the IoU threshold for a given ground truth object, that object is counted as a false negative. Precision is defined as the ratio of true positives to the total number of predicted positives, while recall is the ratio of true positives to the total number of ground truth instances. The mean Average Precision (mAP) summarizes model performance across varying confidence thresholds and IoU values. The definitions of these metrics are as follows Equations 2–4:

(2) $$Precision=\frac{TP}{TP+FP}$$

(3) $$Recall=\frac{TP}{TP+FN}$$

(4) $$mAP=\frac{1}{C}\sum_{c=1}^{C}\left(\frac{1}{N}\sum_{i=1}^{N}AP_{c,i}\right)$$

where C denotes the number of categories, N represents the number of IoU thresholds, and $AP_{c,i}$ is the average precision of the *c*-th category at the *i*-th IoU threshold.

## 3 Experimental results and analysis

### 3.1 Comparative experiment before and after depth filtering

**Figure 11:**
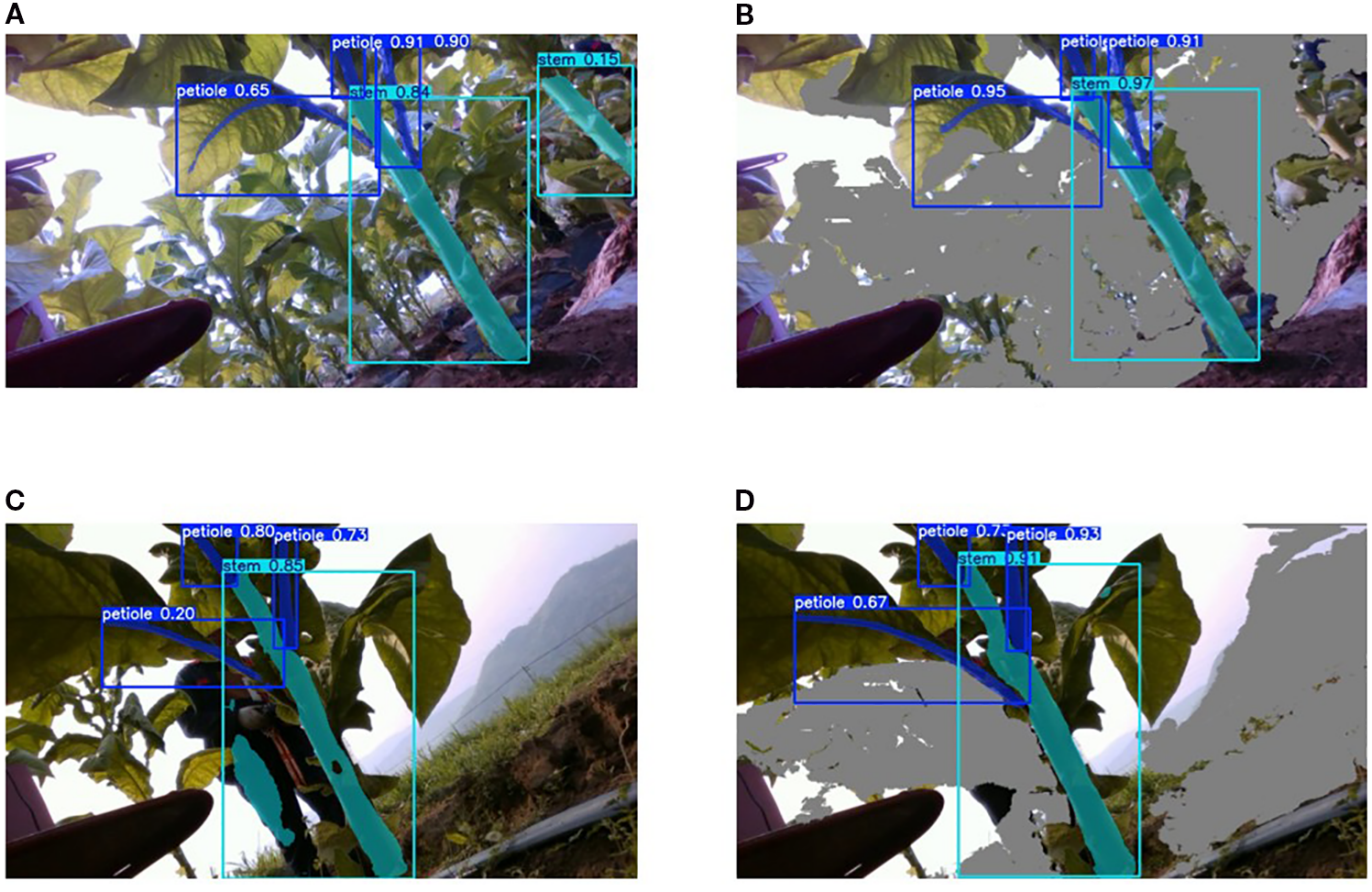
Comparison of segmentation effects. **(A)** before depth filtering. **(B)** after depth filtering. **(C)** before depth filtering. **(D)** after depth filtering.

To evaluate the effectiveness of the proposed depth filtering method for eliminating background interference, a comparative experiment was conducted using two datasets: one with depth filtering applied and one without. Both datasets were evaluated under identical conditions using the same instance segmentation model, YOLOv8-seg.The experimental results are presented in **Figure 11**.

As shown in **Table 1**, applying depth filtering significantly improves segmentation performance under the same model. Specifically, the mAP50^bb^ increases by 7.9%, and the mAP50^seg^ improves by 6.3%. Depth filtering effectively removes background interference, such as distant plants (**Figures 11A, B**), as well as other non-target objects in the background (**Figures 11C, D**), thereby enhancing the accuracy of key part segmentation.

**Table 1:** Comparison of experimental results of different dataset types.

Dataset type	mAP50^bb^/%	mAP50^seg^/%
Before depth filtering	76.4	74.8
After depth filtering	84.3	81.1

### 3.2 Ablation experiments

#### 3.2.1 Comparison of different detection heads

**Table 2** presents the segmentation results obtained using different detection head architectures. As shown in the data, when the DEConv module is not incorporated—i.e., when the detection head is replaced with LSCD—channel sharing across different layers reduces computational cost. However, this simplification results in a slight decline in segmentation performance.

**Table 2:** Ablation study of the detection head.

Head	mAP50^bb^/%	mAP50-95^bb^/%	mAP50^seg^/%	mAP50-95^seg^/%
v8-Head	84.3	51.0	81.1	44.5
LSCD	82.6	49.7	80.2	42.3
LSDECD	86.0	55.1	85.2	47.1

After integrating the DEConv module and upgrading the detection head to LSDECD, the model demonstrates an improvement across all segmentation metrics. This indicates that LSDECD not only preserves computational efficiency through its shared structure but also significantly enhances the model’s ability to capture fine-grained details, thereby leading to superior overall segmentation performance.

#### 3.2.2 C2f-HDRAB embedding location ablation study

**Figure 12:**
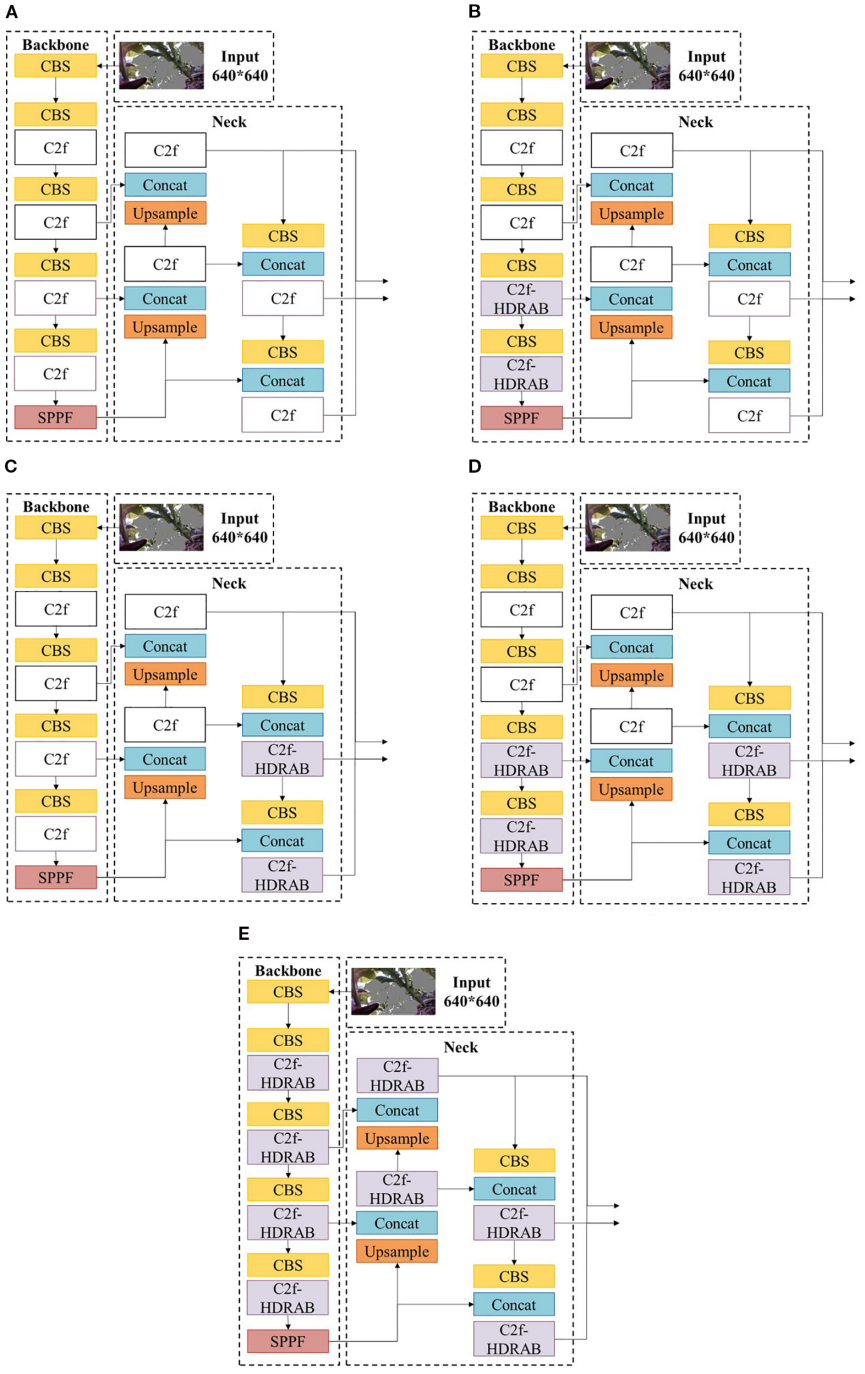
The network structure of the backbone and neck parts in different situations.

Given that HDRAB offers strong structural modeling capabilities but also introduces additional computational overhead, a full-scale replacement of all C2f modules may result in unnecessary performance costs. To systematically investigate the actual impact of HDRAB on segmentation performance, and to evaluate its adaptability and contribution at different network depths, a grouped ablation study was conducted focusing on the replacement positions of the C2f modules. The results are summarized in **Table 3**, where “–” indicates retention of the original C2f structure, and “✓“ denotes substitution with the C2f-HDRAB module. Each row in **Table 3** corresponds to the specific network variants (a–e) illustrated in **Figure 12**.

**Table 3:** Ablation study of c2f-hdrab embedding position.

Shallow Backbone	Deep Backbone	Shallow Neck	Deep Neck	mAP50^bb^/%	mAP50-95^bb^/%	mAP50^seg^/%	mAP50-95^seg^/%	GFLOPs
–	–	–	–	84.3	51.0	81.1	44.5	42.7
–	✓	–	–	86.7	53.1	85.5	44.8	43.1
–	–	–	✓	85.9	52.2	84.6	44.9	43.4
–	✓	–	✓	89.0	55.0	88.3	45.2	43.8
✓	✓	✓	✓	89.2	55.3	88.7	45.5	45.0

As shown in **Table 3**, enhancing the C2f modules with HDRAB in the deeper layers of the backbone and neck improves the model’s segmentation accuracy. When all C2f modules are replaced with C2f-HDRAB, the model achieves the highest segmentation performance, with mAP50^bb^ and mAP50^seg^ reaching 89.2% and 88.7%, respectively. However, when only the deep-layer C2f modules in the backbone and neck are replaced (as in the fifth row of **Table 3**), the mAP50^bb^ and mAP50^seg^ slightly decrease to 89.0% and 88.3%, respectively, while the total computational cost is reduced by 1.2 GFLOPs compared to the full replacement strategy.

These results indicate that substituting C2f modules in shallow layers yields limited performance gains while introducing unnecessary computational overhead. Therefore, we adopt the improvement strategy shown in the fifth row—modifying only the deep layers—is adopted as the final configuration. The corresponding network structure is illustrated in **Figure 12D**.

#### 3.2.3 Ablation experiments on detection head and improved C2f

**Table 4** presents the ablation results for the improved detection head and the enhanced C2f module, along with the mAP scores for petioles and main stems across different model variants. Compared to petiole segmentation, the models consistently demonstrate better performance in detecting and segmenting the main stem. This disparity may be attributed to the irregular growth patterns and complex contours of petioles.

**Table 4:** Ablation study of detection head and C2f.

C2f-HDRAB	LSDECD	Class	mAP50^bb^/%	mAP50-95^bb^/%	mAP50^seg^/%	mAP50-95^seg^/%
–	–	all	84.3	51.0	81.1	44.5
		petiole	74.2	42.6	69.5	29.8
		stem	91.1	54.4	92.2	54.1
✓	–	all	89.0	55.0	88.3	45.2
		petiole	85.5	51.6	82.5	31.7
		stem	92.5	58.5	94.0	58.7
–	✓	all	86.0	55.1	85.2	47.1
		petiole	76.3	48.0	72.2	32.1
		stem	95.7	66.2	97.3	62.2
✓	✓	all	89.5	60.4	91.1	50.8
		petiole	86.0	50.9	85.2	37.8
		stem	94.9	65.6	98.1	63.8

However, in the subsequent task of computing key distances, petioles are primarily used to locate specific key points, and a complete view of the petiole is not necessary. In contrast, the accuracy of the main stem contour is critical, as it directly affects measurement precision. Therefore, the observed segmentation performance for petioles remains acceptable within the context of this application.

The combination of the improved C2f module and the enhanced detection head yields the best overall performance, producing the most significant improvements in both detection and segmentation across all target categories. Compared to the baseline model, mAP50^bb^ and mAP50-95^bb^ increased by 5.2% and 9.4%, respectively, while mAP50^seg^ and mAP50-95^seg^ improved by 10% and 6.3%. The integration of HDRAB into the C2f module enhances the model’s ability to extract and fuse multi-scale features, while the incorporation of the DEConv module into the detection head strengthens fine-detail extraction. The synergy of these two architectural enhancements contributes to the optimal performance of the proposed segmentation model.

### 3.3 Comparison of different models

To further validate the effectiveness of our proposed improvements, the performance of our model was compared against several widely used instance segmentation models on the validation set. YOLOv8-seg, known for its real-time performance, serves as a strong single-stage segmentation baseline. Mask R-CNN, a representative two-stage object detection and segmentation framework, is recognized for its high accuracy and is commonly used as a benchmark model. SOLOv2 is another high-performing segmentation algorithm that achieves precise instance segmentation without relying on an explicit object detection stage. Accordingly, Mask R-CNN, SOLOv2, YOLOv8-seg (baseline), and our improved model were selected for comparative evaluation. Transformer-based segmentation models (e.g., SegFormer, Mask2Former) were also considered during our experimental design. However, the implementations available in MMDetection are typically large-scale models with high memory and computational demands, making them unsuitable for our experimental settings and impractical for edge deployment scenarios. As the primary goal of this work is to enhance YOLOv8 for real-time agricultural applications, rather than to optimize transformer-based frameworks, these models were not included in the current comparative evaluation.

As shown in **Table 5**, Mask R-CNN achieves the highest segmentation performance among all compared models, with mAP50^bb^ and mAP50^seg^ reaching 85.5% and 87.2%, respectively. However, it has the lowest computational efficiency, requiring 131 GFLOPs. YOLOv8-seg demonstrates the highest computational efficiency, with a FLOPs count of only 42.7 GFLOPs, but its segmentation performance is relatively lower, with mAP50^bb^ and mAP50^seg^ at 84.3% and 81.1%, respectively.

**Table 5:** Computational efficiency and segmentation effect of each model.

Model	Size/M	GFLOPs	Times/ms	mAP50 ^bb^/%	mAP50-95^bb^/%	mAP50 ^seg^/%	mAP50-95^seg^/%	Precision ^bb^/%	Recall ^bb^/%	Precision ^seg^/%	Recall ^seg^/%
SOLOv2	137	114	143	–	–	85.4	46.9	–	–	89.5	88.4
Mask R-CNN	335	131	500	85.5	55.2	87.2	47.2	89.6	86.2	91.0	89.6
YOLOv8-seg	12.86	42.7	18	84.3	51	81.1	44.5	87.3	84.1	85.1	82.8
YOLO-HDRAB-LSDECD	12.86	43.8	19	89.5	60.4	91.1	50.8	92.0	89.5	93.7	92.5

In contrast, our improved model achieves the best overall performance, with mAP50^bb^ and mAP50^seg^ reaching 89.5% and 91.1%, respectively. It also obtains the highest precision and recall scores: Precision^bb^ at 92.0%, Recall^bb^ at 89.5%, Precision^seg^ at 93.7%, and Recall^seg^ at 92.5%. Despite this performance, the model maintains a low computational cost of 43.8 GFLOPs, making it well-suited for deployment on edge devices.

**Figure 13:**
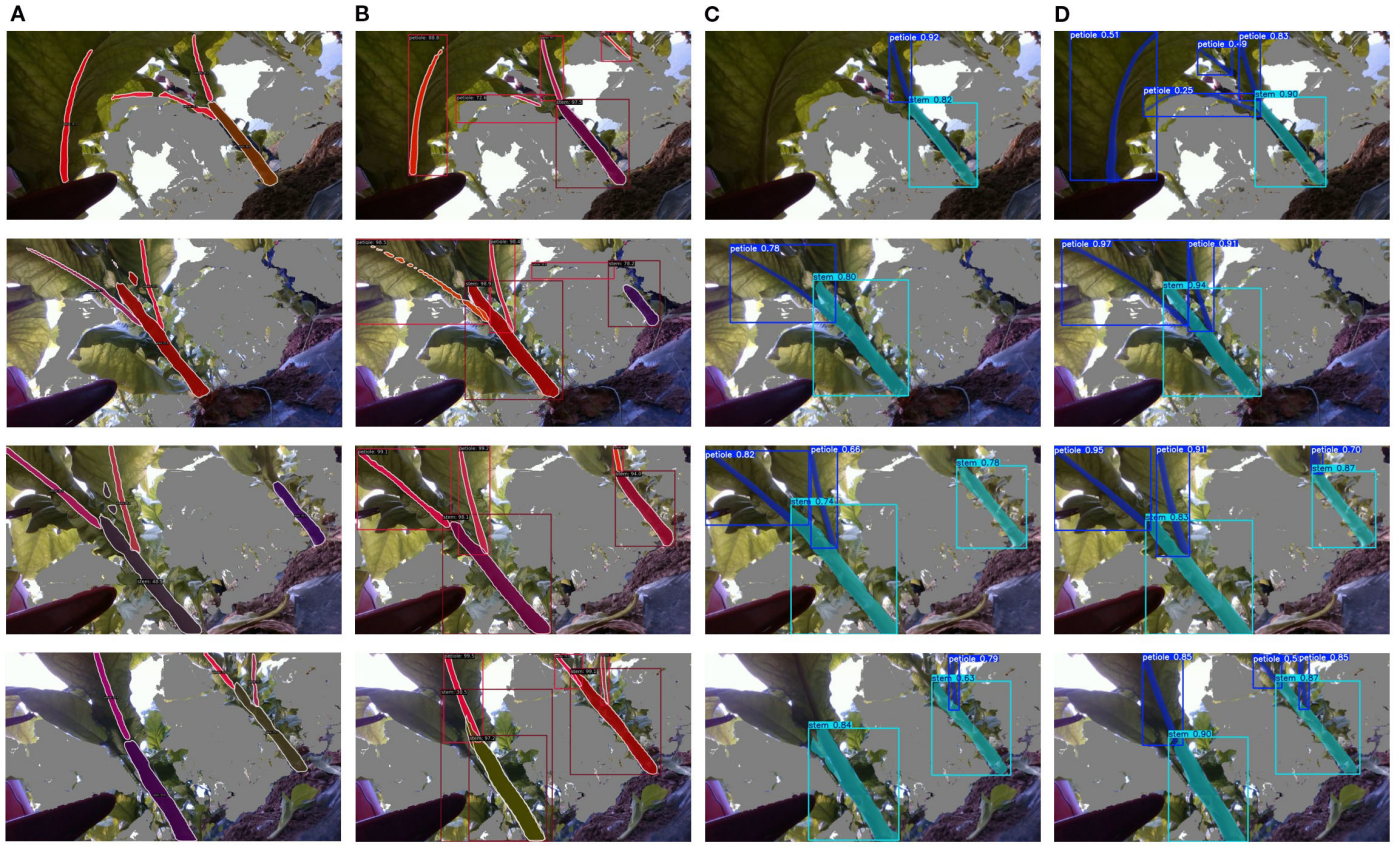
Segmentation results of different algorithms. **(A)** SOLOv2. **(B)** Mask R-CNN. **(C)** YOLOv8-seg. **(D)** YOLO-HDRAB-LSDECD.

**Figure 13** presents the segmentation results on the same image using four models: SOLOv2, Mask R-CNN, YOLOv8-seg, and our improved model, YOLO-HDRAB-LSDECD. As illustrated, our improved model successfully segments all key parts of the tobacco plant, outperforming SOLOv2 and YOLOv8-seg. Mask R-CNN ranks second in terms of segmentation completeness.

A closer comparison between YOLO-HDRAB-LSDECD and Mask R-CNN reveals that Mask R-CNN struggles to accurately distinguish between petioles and main stems, often producing overlapping segmentations. This issue is evident in the 2nd, 3rd, and 4th rows of **Figure 13B**, where petiole–stem overlaps are observed, potentially compromising subsequent calculations of internodal spacing. Moreover, Mask R-CNN tends to generate false positives, as seen on the right side of the 2nd row in **Figure 13B**, further affecting downstream processing.

In contrast, our improved model demonstrates the clearest boundary distinction between petioles and main stems, highlighting the effectiveness of the HDRAB module in addressing boundary ambiguity and improving structural segmentation accuracy.

**Figure 14:**
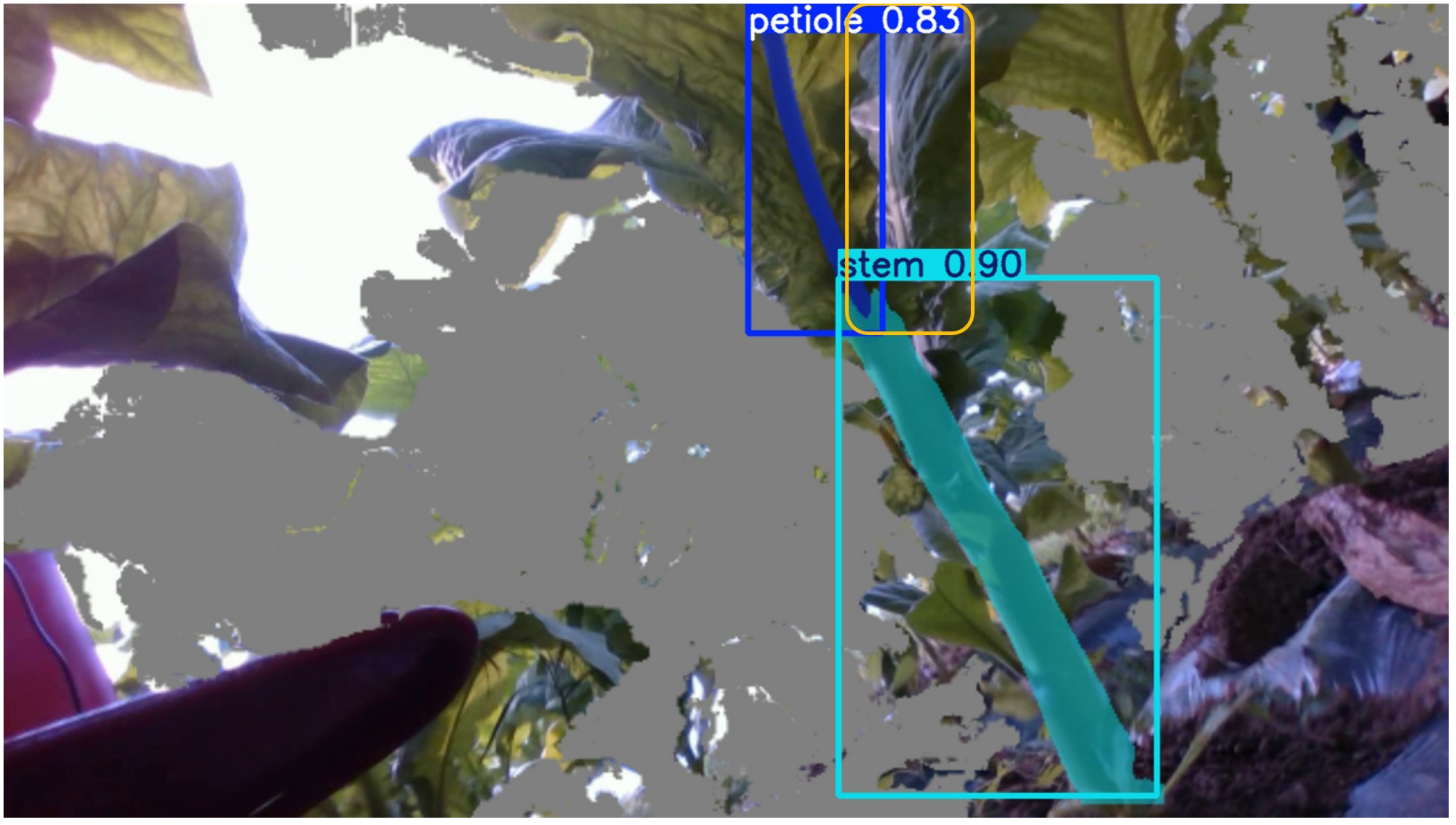
Case of segmentation failure.

In addition to the successful segmentation results, we also present some challenging cases (**Figure 14**). For example, under severe occlusion, one petiole was correctly identified, while another petiole was missed due to leaf occlusion and unfavorable viewing angles, as highlighted by the yellow box in the figure. These failure cases indicate that incorporating more training samples with such occlusion scenarios can further improve the model performance. This will be considered in our future work.

## 4 Discussion

This study addresses the problem of instance segmentation for tobacco plants, specifically targeting the complex structures and blurred boundaries between petioles and main stems. A refined segmentation method based on an improved YOLOv8-seg framework is proposed. To tackle the boundary ambiguity encountered in traditional methods when dealing with intricate plant structures, a Hybrid Dilated Residual Attention Block (HDRAB) is introduced to enhance the model’s capability for joint modeling of local and global information.

Compared with classic methods such as Mask R-CNN (He et al., 2017) and SOLOv2 (Wang et al., 2020), the proposed model demonstrates superior performance in fine-grained boundary segmentation under complex field conditions. Specifically, while Mask R-CNN achieves high overall segmentation accuracy, it frequently produces overlapping detections and false positives in the petiole–stem junction areas, which negatively impacts subsequent internodal distance measurements. Although SOLOv2 circumvents the dependency on object detection, it lacks the capacity for fine-scale texture modeling, resulting in insufficient boundary detail representation.

Yang et al. proposed an improved DeepLabv3+ method incorporating DenseASPP and strip pooling strategies (Jia et al., 2023), achieving a mean Intersection-over-Union (mIoU) of 90.8% in leaf segmentation and exhibiting good boundary handling. However, their approach shows limited effectiveness when applied to plants with complex intertwined branches, such as tobacco. Similarly, Li et al. developed PSegNet (Li et al., 2022), which achieves robust segmentation performance in plant point cloud data, particularly in distinguishing leaf and stem structures. Nevertheless, point cloud acquisition is costly and less practical for real-time field applications.

In contrast, the YOLO-HDRAB-LSDECD model proposed in this study, operating on two-dimensional RGB-D images, combined with a depth-based background filtering strategy, significantly improves foreground segmentation quality and effectively suppresses background vegetation interference, providing a more reliable foundation for subsequent structural analysis and growth metric extraction.

In terms of detailed feature modeling, the introduced Detail-Enhanced Convolution (DEConv) module leverages multi-directional gradient differences to enhance sensitivity to local high-frequency features. Compared to existing hybrid vision networks that combine keypoint detection with segmentation (Li et al., 2024), the proposed approach achieves mAP50^bb^ and mAP50^seg^ scores of 89.5% and 91.1%, respectively, surpassing existing unimodal methods in terms of fine-grained boundary delineation, all while maintaining low computational overhead.

Despite these promising results, several limitations and areas for further investigation remain. Firstly, the current approach primarily relies on RGB-D sensors; however, depth information can be easily affected by strong illumination or weak reflectivity, potentially resulting in the loss of key petiole points. Future work could explore the integration of multi-modal sensor data, such as hyperspectral or thermal imaging, combined with RGB-D data (Kim and Chung, 2021), to enhance robustness under complex lighting conditions.

Furthermore, the architectural components introduced in this work were selected with specific challenges in mind. HDRAB was designed to enlarge the receptive field while retaining spatial resolution, thereby enhancing the discrimination of blurred boundaries at the stem–petiole junctions. DEConv was introduced to capture multi-directional gradient information, strengthening fine-grained feature extraction under occlusion. Although exhaustive comparisons with other attention modules such as SE or CBAM were not included, the ablation experiments (Section 3.2) demonstrate the effectiveness of the chosen designs. Future studies will further investigate alternative lightweight attention mechanisms to provide a broader comparative analysis.

In addition, the current dataset was collected in a relatively limited geographical location and cultivation environment, which may restrict the generalization ability of the model. Future work will focus on expanding the dataset to include tobacco plants from multiple regions, varieties, and growth stages, enabling more comprehensive validation and further improving the robustness and adaptability of the proposed approach. Beyond tobacco, the proposed framework also has the potential to be adapted to other crops with similar structural segmentation challenges, and future validation on species such as tomato or cucumber will help further examine its generalizability.

Although the proposed method maintains good inference efficiency under limited computational resources, further optimization in model compression and acceleration is necessary to enable broader real-time field deployment. In addition, lightweight transformer-based segmentation architectures, once they become more mature and efficient, could also be investigated as complementary baselines to provide a broader comparison. Quantitative analysis of model inference speed will also be carried out, with potential exploration into lightweight transformer architectures or acceleration techniques based on pruning and quantization, aiming to reduce the deployment barrier.

In conclusion, this study proposes an effective method for the instance segmentation of key structural components in tobacco plants. The approach achieves superior performance in boundary ambiguity handling and fine-grained texture extraction compared to existing methods, validating the effectiveness of the proposed module designs and providing a solid foundation for future research in plant structure recognition and intelligent perception applications.

## 5 Conclusions

This study addresses the task of segmenting key structural components of tobacco plants by proposing an enhanced instance segmentation method based on the YOLOv8-seg framework. Considering the complexity of field environments, targeted improvements were introduced at both the network architecture and data preprocessing levels.

Specifically, a depth-based background filtering strategy was employed to suppress interference from non-target vegetation, significantly enhancing foreground segmentation quality. The backbone was augmented with a Hybrid Dilated Residual Attention Block (HDRAB) to strengthen multi-scale contextual feature extraction, while the detection head was redesigned into a lightweight LSDECD structure incorporating a Detail-Enhanced Convolution (DEConv) module to improve the representation of fine textures and boundary details.

Experimental results demonstrate that the introduction of the depth filtering mechanism improves mAP50^bb^ from 76.4% to 84.3% and mAP50^seg^ from 74.8% to 81.1%, achieving gains of 7.9% and 6.3%, respectively, thereby validating its effectiveness in mitigating background noise. Furthermore, architectural enhancements through HDRAB and LSDECD boost mAP50^bb^ and mAP50^seg^ to 89.5% and 91.1%, corresponding to improvements of 5.2% and 10.0% over the baseline YOLOv8-seg model.

Compared to mainstream instance segmentation models such as Mask R-CNN and SOLOv2, the proposed method demonstrates significant advantages in both segmentation accuracy and computational efficiency. Specifically, Mask R-CNN achieves mAP50^bb^ and mAP50^seg^ scores of 85.5% and 87.2% on the validation set but incurs a high computational cost of 131 GFLOPs, making it unsuitable for real-time deployment in field conditions. SOLOv2, while eliminating dependency on an explicit detection stage, attains an mAP50^seg^ of 85.4%, but still falls short in fine-grained boundary delineation. YOLOv8-seg, as a single-stage baseline model, achieves the highest inference efficiency with only 42.7 GFLOPs; however, its segmentation accuracy is relatively lower, with mAP50^bb^ and mAP50^seg^ scores of 84.3% and 81.1%, respectively.

In contrast, the proposed YOLO-HDRAB-LSDECD model achieves a better balance between segmentation accuracy and efficiency, reaching mAP50^bb^ and mAP50^seg^ scores of 89.5% and 91.1%, respectively. Furthermore, it attains Precision^bb^ and Recall^bb^ scores of 92.0% and 89.5%, and Precision^seg^ and Recall^seg^ scores of 93.7% and 92.5%, respectively, while maintaining a low computational cost of just 43.8 GFLOPs. This method not only significantly improves segmentation accuracy in regions with blurred boundaries but also maintains low computational complexity, demonstrating excellent potential for deployment in real-world agricultural field environments.

## Data Availability

The raw data supporting the conclusions of this article will be made available by the authors, without undue reservation.
