# Correction: An improved YOLOv8-seg-based method for key part segmentation of tobacco plants

**DOI:** 10.3389/fpls.2026.1776055

**Published:** 2026-02-24

**Authors:** Yihao Liu, Du Chen, Yawei Zhang, Xin Wang

**Affiliations:** 1 College of Engineering, China Agricultural University, Beijing, China; 2 State Key Laboratory of Intelligent Agricultural Power Equipment, Beijing, China

**Keywords:** YOLOv8, deep learning, agricultural robots, tobacco harvesting, automated harvesting

An incorrect **Funding** statement was provided. The incorrect **Funding** statement reads: “The author(s) declare financial support was received for the research and/or publication of this article. This work was supported by the Horizontal Research Project of China Agricultural University (Project No. 202405410711069).” The correct sentence reads: “The author(s) declare financial support was received for the research and/or publication of this article. This work was supported by the Key Research and Development Program of China National Tobacco Corporation, titled “Key Technologies Research and Development for Intelligent Tobacco Leaf Harvesting” (Grant No. 110202301016). The authors declare that this study received funding from China National Tobacco Corporation. The funder was not involved in the study design, collection, analysis, interpretation of data, the writing of this article or the decision to submit it for publication.”.

The original version of this article has been updated.

